# Walking characteristics including mild motor paralysis and slow walking speed in post-stroke patients

**DOI:** 10.1038/s41598-020-68905-3

**Published:** 2020-07-16

**Authors:** Naomichi Mizuta, Naruhito Hasui, Tomoki Nakatani, Yusaku Takamura, Shintaro Fujii, Masako Tsutsumi, Junji Taguchi, Shu Morioka

**Affiliations:** 1 Department of Neurorehabilitation, Graduate School of Health Sciences, Kio University, 4-2-2 Umaminaka, Koryo, Kitakatsuragi-gun, Nara, 635-0832 Japan; 2 Department of Therapy, Takarazuka Rehabilitation Hospital, Medical Corporation SYOWAKAI, 22-2 Tsurunoso, Takarazuka-shi, Hyogo 665-0833 Japan; 3 Neurorehabilitation Research Center, Kio University, 4-2-2 Umaminaka, Koryo, Kitakatsuragi-gun, Nara, 635-0832 Japan

**Keywords:** Neuroscience, Medical research, Neurology

## Abstract

Walking speed is strongly influenced by the severity of motor paralysis in post-stroke patients. Nevertheless, some patients with mild motor paralysis still walk slowly. Factors associated with this difference in walking speed have not been elucidated. To confirm walking characteristics of patients with mild motor paralysis and slow walking speed, this study identified patient subgroups based on the association between the severity of motor paralysis and walking speed. Fugl-Meyer assessment synergy score (FMS) and the walking speed were measured (n = 42), and cluster analysis was performed based on the association between FMS and walking speed to identify the subgroups. FMS and walking speed were associated (ρ = 0.50); however, some patients walked slowly despite only mild motor paralysis. Cluster analysis using FMS and walking speed as the main variables classified patients into subgroups. Patients with mild motor paralysis (FMS: 18.4 ± 2.09 points) and slow walking speed (0.28 ± 0.14 m/s) exhibited poorer trunk stability, increased co-contraction of the shank muscle, and increased intramuscular coherence in walking compared to other clusters. This group was identified by their inability to fully utilize the residual potential of motor function. In walking training, intervention in instability and excessive cortical control may be effective.

## Introduction

The characteristics of walking disturbances in post-stroke patients often include reduced walking speed, step length, trunk stability, symmetry (compensation strategy), and automatic walking control. It is important to note that walking speed is strongly associated with fall risk, quality of life, and walking independence^1–3^, and thus the importance of rehabilitation intended for the promotion of walking speed has been widely recognized^2,4^. It has been found that the propulsion force of the paralyzed side during the push-off phase is a primary contributor to important biomechanical factors^5,6^.

The trailing limb angle affected the production of propulsion force^7^. Trailing limb angle indicates the propulsion vector^8^, and previous studies have shown that a decrease in this angle was considerably related to a decrease in walking speed in post-stroke patients^7,9^. In addition, deviation was not restricted to the kinematics pattern of the leg in the paralyzed side alone. Trunk instability due to reduced balance ability was also noted and was associated with decreased walking speed^10^. The deviation in the kinematic factors have been reported to cause differences in the muscle activity pattern in the paralyzed side during walking^11^. Specifically, the co-contraction of the shank muscles was observed to increase in the stance phase, and the limited ankle rocker function in the stance phase produced a strong influence on walking speed^12^. It is important to note that the kinematic characteristics of walking disorders may be strongly influenced by central neurological deficits. Therefore, both kinetic and neurological factors need to be considered^13^. Neurological factors that affect walking speed include motor paralysis, sensory disorder, and muscle spasticity, leading to a decrease in stride length and trunk stability and an increase in asymmetry, all of which can limit walking speed^14–16^. Among neurological findings, the excitability of the corticospinal tract is strongly influenced by leg muscle activity during walking^17^. In addition, corticospinal tract activity during walking is substantially reduced post-stroke, which clearly decreases the walking speed^18^.

Although the severity of motor paralysis strongly affects walking speed post-stroke, there are some clinical cases in which walking speed is slow despite only mild motor paralysis^15,19^. The fact that walking speed is slow even in cases of mild motor paralysis indicates that the residual function cannot be determined by the severity of motor paralysis alone. Previous studies have shown that voluntary motor function and muscle activity intensity differ during walking^19^. Therefore, we considered that motor paralysis evaluation at rest does not always reflect walking performance. This may be explained by the presence of other factors, such as balance, compensatory strategies, muscle activity patterns, and the dependence of voluntary or automatic walking control^20,21^. Previous studies have comprehensively investigated the walking characteristics of stroke patients, however, have not analyzed subgroups based on the association between the severity of motor paralysis and walking speed. In the absence of subgroup analysis, the differences in factors limiting walking speed may not be apparent. Therefore, to analyze the walking characteristics of patients with mild motor paralysis and slow walking speed, cluster analysis is necessary. We hypothesized that in patients with mild motor paralysis and slow walking speed, the characteristics of walking instability, increased co-contraction of the shank muscles, and excessive voluntary control may affect slow walking speed. In this study, we identified groups of post-stroke patients with mild motor paralysis and slow walking speed using cluster analysis to be able to determine the association between the severity of motor paralysis and walking speed. In addition, the characteristics of walking disturbances in patients with mild motor paralysis and slow walking speed were examined using kinematics and electromyography (EMG). A comprehensive examination of factors associated with walking speed and the identification of limiting factors for walking speed in patients with mild motor paralysis and slow walking speed are important for the promotion of effective rehabilitation.

## Methods

### Participants

Forty-two post-stroke patients (mean ± standard deviation, 65.9 ± 13.7 years; stroke onset, 132.1 ± 107.6 days) were enrolled at the Takarazuka Rehabilitation Hospital of Medical Corporation SYOWAKAI, in this cross-sectional study (see Table 1 for demographic information). The exclusion criteria set were : (1) cannot walk independently without assistance of physical therapists, (2) inability to walk without using walking aids with casters, (3) bilateral lesions, (4) a passive range of < 5° of hip joint extension in the paralyzed side and an ankle dorsiflexion range of < 0° at the knee joint in the complete extension position on the paralyzed side, (5) Mini-Mental State Examination score < 24 points, (6) history of orthopedic disease, (7) did not have pain, (8) cerebellar lesions or resting tremors, and (9) unilateral spatial neglect, except stroke. Measurements were performed continuously on participants who cleared the exclusion criteria. All participants provided informed consent prior to the beginning of the study. All procedures were approved by the ethics committee of Takarazuka Rehabilitation Hospital of Medical Corporation SYOWAKAI (ethics review number; 20170201) and were in accordance with the Declaration of Helsinki.

**Table 1:** Characteristics of the subjects.

	All subjects (n = 42)	Cluster 1 (n = 9)	Cluster 2 (n = 5)	Cluster 3 (n = 7)	Cluster 4 (n = 10)	Cluster 5 (n = 11)
Age (years)	65.9 ± 13.7	72.4 ± 12.5	63.6 ± 14.5	52.6 ± 5.94	69.4 ± 5.89	67.0 ± 18.0
Sex (n): male/female	34/8	8/1	3/2	7/0	8/2	8/3
Affected side (n): right/left	26/16	6/3	1/4	6/1	8/2	6/5
Time since stroke (days)	132.1 ± 107.6	180.4 ± 178.2	176.6 ± 130.0	123.7 ± 65.5	105.8 ± 57.8	101.4 ± 69.6
Functional ambulation category	3.57 ± 0.74	3.0 ± 0.0	3.20 ± 0.45	3.43 ± 0.54	3.70 ± 0.68	4.18 ± 0.87
Using assist device (n): no use/T-cane/Q-cane	36/4/2	4/4/1	4/0/1	7/0/0	10/0/0	11/0/0
FMS (lower extremity): max = 22*	17.5 ± 4.53	18.4 ± 2.09	11.6 ± 1.81	12.1 ± 1.57	20.4 ± 1.51	21.9 ± 0.30
FMA sensory score (lower extremity): max = 12^†^	8.82 ± 2.96	8.44 ± 1.88	6.60 ± 3.29	10.1 ± 2.85	8.5 ± 3.66	9.18 ± 2.86
Modified Ashworth Scale: max = 5^‡^	1.11 ± 1.04	1.89 ± 0.78	1.60 ± 1.52	1.57 ± 0.79	0.67 ± 0.71	0.27 ± 0.65
Waking speed (m/s)	0.74 ± 0.33	0.28 ± 0.14	0.48 ± 0.15	0.86 ± 0.09	0.84 ± 0.09	1.08 ± 0.11
Symmetry index in SS (%)^§^	-75.8 ± 22.6	-106.7 ± 18.1	-91.3 ± 15.5	-65.5 ± 6.31	-62.1 ± 4.79	-54.3 ± 7.93
Trailing limb angle (°)	8.53 ± 6.72	0.16 ± 4.91	2.33 ± 4.65	10.0 ± 2.14	10.9 ± 3.13	13.9 ± 2.16

The data are reported as the mean ± standard deviation or n.

*Synergy score of the Fugl-Meyer assessment (FMS).

^†^Sensory score of the Fugl-Meyer assessment (FMA).

^‡^To evaluate the spasticity of the ankle plantar flexor muscle, a Modified Ashworth Scale was used and evaluated on a 0–5 scale.

^§^If the symmetry index was low, the paralyzed side in the single-leg support phase was shorter than the non-paralyzed side.

### Experimental set-up and procedures

Participants were asked to walk five times at a comfortable speed on a 10-m walkway with a supplementary 2-m walkway, assisted by a physical therapist nearby to eliminate the risk of falling. Participants were allowed to use a cane as necessary during assessments. An ankle foot orthosis with an oil damper, which was necessary for the use of measuring instruments (Gait Judge System: Pacific Supply, Japan), was attached to the paralyzed lower limb side^22,23^. The oil damper unit is located on the lateral side of the ankle joint in such orthoses (Gait Solution Design; Kawamura Gishi, Osaka, Japan)^23^. A small hydraulic cylinder is inserted in the oil damper unit to provide resistance to plantar flexion as needed. The resistive force of the oil damper can be easily changed by adjusting a screw, and the level of kinetics interference to the lower leg becomes very small when the resistance force is adjusted to the minimum setting. In this study, the setting of the resistive force was minimized so as not to affect walking. Wireless tri-axial accelerometers (Gait Judge System: Pacific Supply, Osaka, Japan; sampling rate: 1 kHz) were attached to the back of the third lumbar level. In order to minimize the mixing of components in different coordinate directions in the accelerometer, the initial angle of the vertical axis of the accelerometer was set to coincide with the vertical direction in absolute space. Wireless surface EMG (Gait Judge System: Pacific Supply, Osaka, Japan; sampling rate: 1 kHz) was recorded of tibialis anterior (TA) and medial gastrocnemius (MG) from the paralyzed side. Each skin site was shaved and cleaned with alcohol before electrode placement. To avoid the influence of electronic crosstalk, the distance between the electrodes was set to 20 mm. After removing the initial acceleration and the final deceleration phases from each walking session, 10–13 strides for each participant were extracted. We confirmed that the modified Borg scale was < 4 before the walking measurement in order not to affect walking performance by the fatigue.

### Clinical evaluation

To measure the severity of motor paralysis and sensory disturbances, the lower limb motor score and sensory score from the Fugl-Meyer assessment (FMA) were used^24^. The FMA Synergy score (FMS) was used to determine the FMA motor score^19,25^. To evaluate muscle spasticity of the ankle plantar flexor muscle, the modified Ashworth Scale was used and converted to a 0–5-point^26^.

### Data recording and analysis

The walking speed was measured by a stopwatch when participants passed the start and end lines of the 10-m walk way using recorded video data^27^. To examine compensation strategies during walking, the asymmetry of the single-leg support time of both legs was calculated using a symmetry index^28^. The trailing limb angle was measured using a video camera fixed on the sagittal plane, and analysis timing was set at the moment of heel contact of the non-paralyzed leg^7^. Trailing limb angle was defined as the angle between the vertical axis and the vector joining the greater trochanter with the fifth metatarsal head. After the video of the heel contact timing was converted into a still image, trailing limb angle was calculated using image analysis software (Image J: National Institutes of Health, version 1.48) with markers previously affixed to the greater trochanter and the fifth metatarsal head^7^. In addition, a vertical axis reference frame was placed at a position that did not interfere with the participants' walking. For quantification of trunk instability, signals were obtained from a tri-axial accelerometer attached to the third lumbar level, and subtraction mean value, a low pass filter (Cut off: 10 Hz) and the root mean square waveform^29^. Since the acceleration value is proportional to the square of the velocity, the acceleration signal was corrected by dividing it by the squared value of walking speed^29^. Because the vertical component depends on the walking speed, trunk instability was measured as the sum of the anterior–posterior and lateral components^30^. The auto correlation (AC) of the trunk acceleration was used for trunk motility regularity. AC analysis results changed depending on the time taken for one stride, and the correlation with the original gait cycle was calculated. Moreover, AC used an unbiased method independent of the number of measured data. The higher the value of AC, the better the walking regularity. The skin was sufficiently cleaned with alcohol to reduce impedance, and electrodes were affixed on TA and MG on the paretic side. Raw EMG signals applied band-pass filtered using a zero-lag 4th-order Butterworth filter with cutoff frequencies of 20–450 Hz (use 5–450 Hz for intramuscular coherence analysis), subtraction mean, full-wave rectified. First, in order to characterize the gait phase depending on amplitude and timing changes in the EMG activity, we adapted moving root mean square filter with a 50 ms time window to the obtained EMG envelope and liner interpolation over individual gait cycles to fit a normalized 100-point time base. All pre-processing of EMG was based on the Surface EMG for the Non-Invasive Assessment of Muscles Guidelines (https://www.seniam.org). EMG normalization was divided by the maximal amplitude during walking^31^. The co-contraction index (CI) was calculated as the overlapping rate between TA and MG from the normalized EMG waveform^32^. Intramuscular wavelet coherence analysis (Morlet) was conducted to influence the differences in cortical driven during walking^33^. Wavelet coherence was determined a measure of the correlation between two signals. We defined the wavelet coherence of two variables x and y as follows:

1 $$ {\text{C}}_{{{\text{xy}}}} ({\text{a}},\;{\text{b}})^{2} = \frac{{\left| {{\text{S}}\left( {{\text{C}}_{{\text{x}}}^{*} ({\text{a}},\;{\text{b}}){\text{C}}_{{\text{y}}} ({\text{a}},\;{\text{b}})} \right)} \right|^{2} }}{{{\text{S}}\left( {\left| {{\text{C}}_{{\text{x}}} ({\text{a}},\;{\text{b}})} \right|^{2} {\text{S}}\left( {\left| {{\text{C}}_{{\text{y}}} ({\text{a}},\;{\text{b}})} \right|} \right)} \right)^{2} }} $$

where Cx (a, b) and Cy (a, b) denote the continuous wavelet transforms of x and y at scale a and position b. * indicates the complex conjugate, and S was a smoothing operator in time and scale^34,35^.

Coherence can range from 0 to 1, with 1 showing a perfect linear correlation. Because the coherence of the beta band (15–30 Hz) was strongly reflected in corticospinal tract activity^18^, we calculated beta band mean value in each gait cycle. Identification of the walking events was performed based on the vertical component of the tri-axial accelerometer attached to the third lumbar level and anterior–posterior component of tri-axial accelerometer attached to the shank on the paretic side^36^. Acceleration signals, EMG activities, CI, and wavelet coherence were time-normalized to 100 points of the paralyzed side gait cycle and separately calculated for the first and second double support, single-leg support, and swing phases on the paretic side. MATLAB R2016a (The MathWorks, Inc., Natick, MA, USA) was used for all data analysis.

### Statistical analysis

Spearman’s rank correlation coefficient was determined to confirm the distribution of FMS and comfortable walking speed. Hierarchical cluster analysis (Ward, Mahalanobis’ distance) was performed using FMS and walking speed to identify subgroups according to the distribution between the FMS and walking speed. The optimal number of clusters was determined using gap values^37^. Subsequently, the Kruskal–Wallis test was used to compare all variables and post-hoc Steel–Dwass test to compare the differences between groups. Statistical analyses were performed using the statistical software package R (Ver.3.3.0, R Core Team, 2016). Statistical significance was accepted at p < 0.05.

## Results

### Relationship between the severity of motor paralysis and walking speed

**Figure 1:**
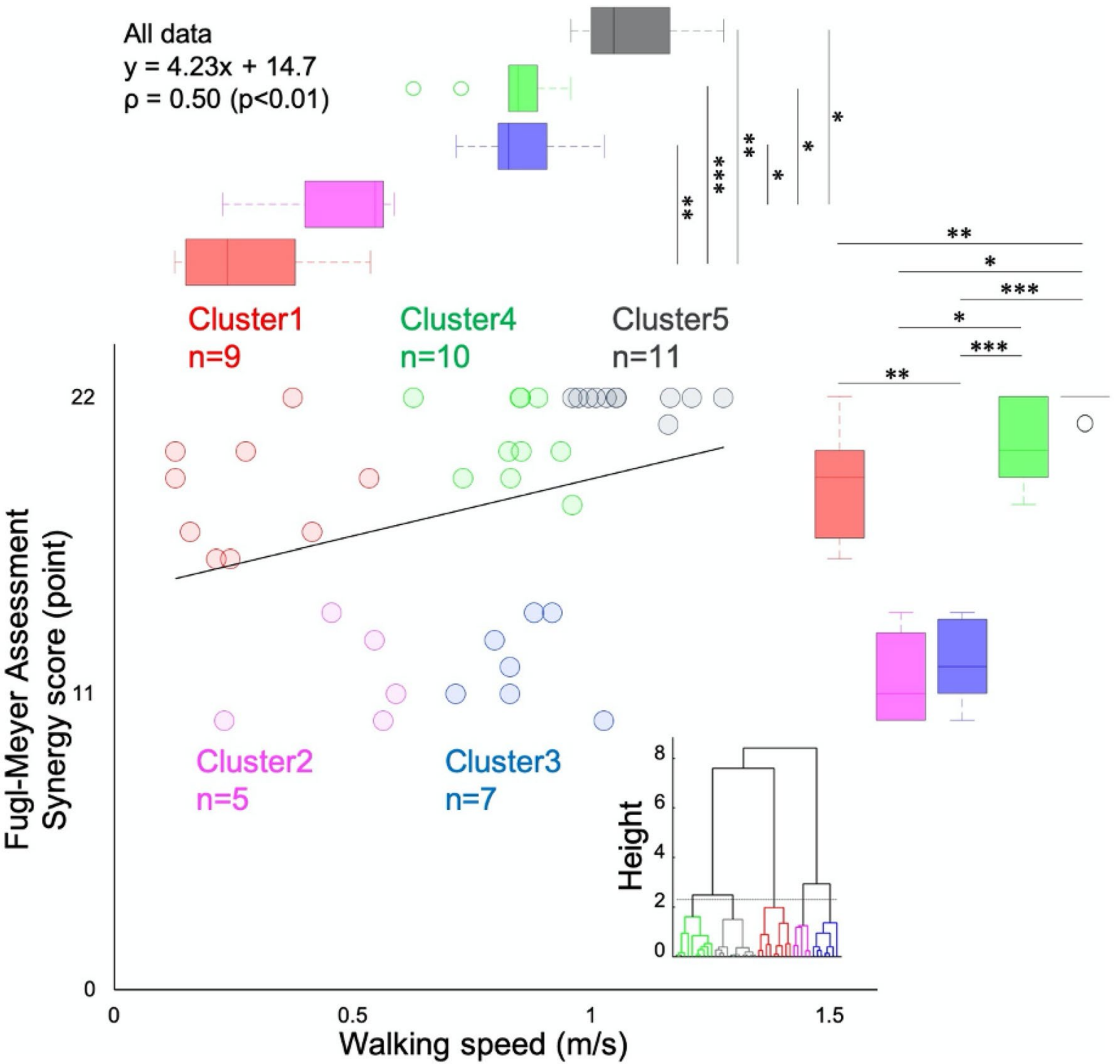
Association between the severity of motor paralysis and walking speed. Distribution of the Fugl-Meyer assessment synergy scores and walking speed in each cluster and dendrogram by hierarchical cluster analysis. Line plot is a regression line of all the data. Although the Fugl-Meyer assessment synergy score and walking speed showed a positive correlation, there were cases in which walking speed was different despite the same motor paralysis severity level. As a result of cluster analysis, five distinct clusters were identified. *p < 0.05, **p < 0.01, ***p < 0.001.

Although FMS and walking speed showed a significant correlation (ρ = 0.50, p < 0.01), in scatter plot between FMS and walking speed, some patients showed slower/faster walking speed with mild/severe motor paralysis. Therefore, the cluster analysis using FMS and walking speed identified five clusters (Fig. 1). FMS showed lower values in clusters 2 (mean ± standard deviation, 11.6 ± 1.81 points) and 3 (12.1 ± 1.57 points) than in other clusters. On the other hand, clusters 1 (18.4 ± 2.09 points) and 4 (20.4 ± 1.51 points, p = 0.25) or clusters 2 and 3 (p = 0.68) in FMS were no significant differences. Walking speed between the clusters showed a increasing tendency from clusters 1 to 5; clusters 1 (0.28 ± 0.14 m/s) and 2 (0.48 ± 0.15 m/s) showed significantly lower walking speeds than clusters 3 (0.86 ± 0.09 m/s, p < 0.01, p < 0.05), 4 (0.84 ± 0.09 m/s, p < 0.001, p < 0.05), and 5 (1.08 ± 0.11 m/s, p < 0.01, p < 0.05). From these results, cluster 1 had mild-to-moderate motor paralysis and poor walking speed, cluster 2 had severe motor paralysis and poor walking speed, cluster 3 had severe motor paralysis and moderate walking speed, cluster 4 had mild-to-moderate motor paralysis and moderate walking speed, and cluster 5 had very slight motor paralysis and fast walking speed. The characteristics of each subgroup are detailed below.

### Clinical evaluation and walking characteristics

**Figure 2:**
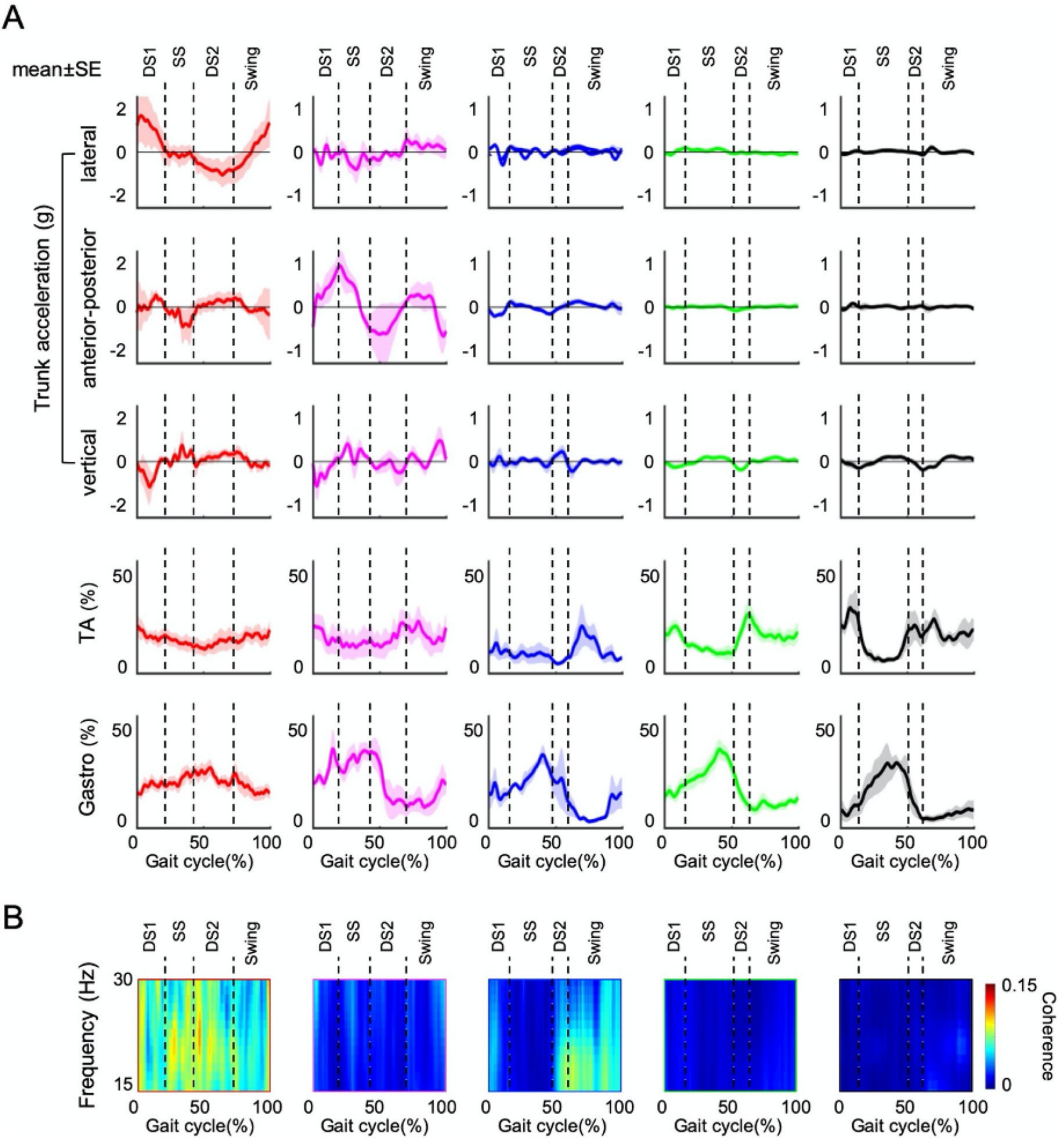
Time-Series data of trunk acceleration and electromyography in each cluster. From the top of (**A**), the trunk acceleration of lateral, anterior–posterior, vertical component, TA, and MG muscle activity are shown for each cluster. Clusters 1–5 are shown from the left. Please note that the trunk acceleration in the Y-axis range was only − 2 to 2 for cluster 1, and − 1 to 1 for clusters 2, 3, 4, and 5. Trunk instability showed high values in clusters 1 and 2 in the anterior–posterior and lateral components during the stance phase. The electromyography waveform of TA activity in cluster 1 and 2 showed little change in muscle activity according to each phase of the gait cycle. (**B**) Shows wavelet coherence of the beta range. The colormap shows the coherence value. Coherence greatly varies among clusters, with cluster 1 having the highest coherence. *SE* standard error, *DS1* first double support phase, *DS2* second double support phase, *SS* single-leg support phase, *Swing* swing phase, *TA* tibialis anterior, *MG* medial gastrocnemius.

**Figure 3:**
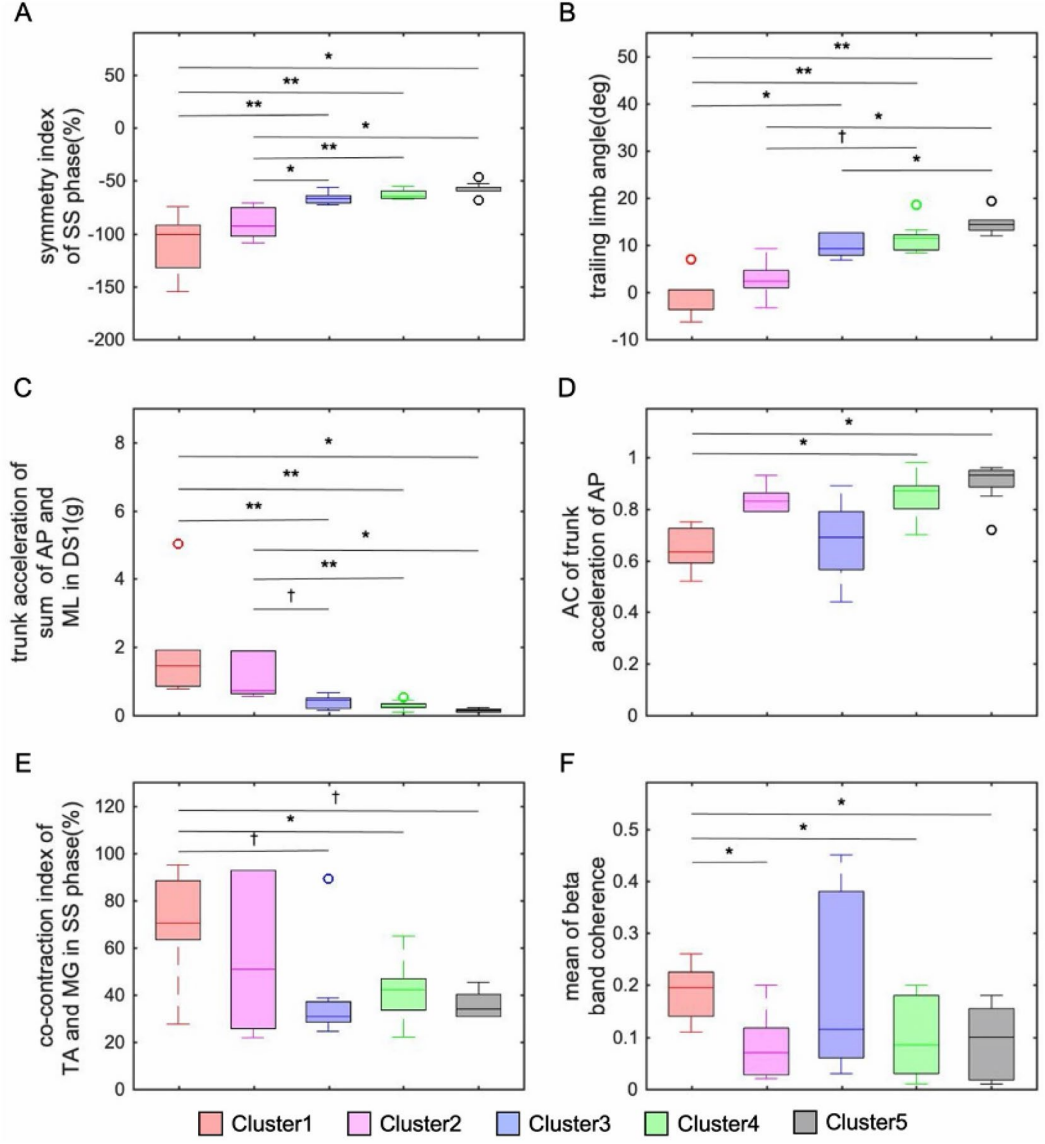
Comparison of walking characteristics in each cluster. The symmetry index of SS time (**A**), trailing limb angle (**B**), the total value of the AP and ML components of trunk acceleration (**C**), the AC of trunk acceleration of AP component (**D**), co-contraction index in the SS phase (**E**), and the coherence of the beta band (**F**) are shown for each cluster, starting on the left with cluster 1. The trailing limb angle among clusters was confirmed to show an increasing value from cluster 1 to 5. ^†^p < 0.10, *p < 0.05, **p < 0.01. *SS* single-leg support phase, *AP* anterior–posterior, *ML* lateral, *AC* auto correlation, *TA* tibialis anterior, *MG* medial gastrocnemius.

The Modified Ashworth Scale of the plantar flexor muscle in clusters 1, 2, and 3 was significantly higher than that in clusters 4 and 5. In contrast, days after stroke onset (p = 0.59) and FMA sensory score were not significantly different for each cluster (p = 0.38). Symmetry index showed steadily increasing values from clusters 1 to 5 (Fig. 3A). The trailing limb angle increased from cluster 1 to 5, and cluster 1 (0.16 ± 4.91 degree) showed lower than clusters 3(10.0 ± 2.14 degree, p < 0.05), 4 (10.9 ± 3.13 degree, p < 0.01), and 5 (13.9 ± 2.16 degree, p < 0.01). Cluster 2 (2.33 ± 4.65 degree) had a significantly lower angle than cluster 5 (p < 0.05) and had a tendency of lower angle than cluster 4 (p = 0.06, Fig. 3B). Trunk acceleration was high values in clusters 1 and 2 in the anterior–posterior and lateral components in first and second double support phases (Fig. 2A). Trunk acceleration (total of anterior–posterior and lateral components) in first double support phase was significantly higher in cluster 1 (1.69 ± 1.34) than that in cluster 3 (0.39 ± 0.20, p < 0.01), 4 (0.28 ± 0.13, p < 0.01), and 5 (0.15 ± 0.05, p < 0.05). Cluster 2 (1.07 ± 0.63) had a significantly higher trunk acceleration than cluster 4 (p < 0.01) and 5 (p < 0.05) and tended to have higher trunk acceleration than cluster 3 (p = 0.09, Fig. 3C). The AC of the anterior–posterior trunk acceleration was significantly lower in cluster 1 (0.67 ± 0.11) than in cluster 4 (0.86 ± 0.08, p < 0.05) and 5 (0.89 ± 0.09, p < 0.05). Moreover, AC of the anterior–posterior component was significantly lower in cluster 3 (0.75 ± 0.11) than in cluster 5 (p < 0.05, Fig. 3C). The AC of the vertical trunk acceleration was significantly lower in cluster 1 (0.49 ± 0.18) than that in clusters 4 (0.87 ± 0.06, p < 0.01) and 5 (0.91 ± 0.08, p < 0.01). Additionally, AC of the vertical trunk acceleration was significantly lower in cluster 3 (0.74 ± 0.11) than in cluster 4 (p < 0.05) and 5 (p < 0.05, Fig. 3D). However, the AC of the lateral trunk acceleration demonstrated no significant difference in each cluster. Average amplitude of TA and MG activities showed no significant differences in all gait sub-phases. EMG waveforms of TA and MG demonstrated less change in muscle activity according to each sub-phase of the gait cycle in clusters 1 and 2, and the overlapping area showed a different in each cluster and gait cycle (Fig. 2A). Especially, CI in the single-leg support phase was significantly higher in cluster 1 (72.2% ± 21.8%) than in cluster 4 (41.4% ± 12.0%, p < 0.05, Fig. 3E). Furthermore, CI of cluster 1 had a tendency to show a higher than clusters 3 (37.8% ± 19.8%, p = 0.10) and 5 (35.8% ± 5.76%, p = 0.09, Fig. 3E). The wavelet coherence of the beta band was different trends depending on the timing of gait cycle (Fig. 2B). The coherence of the beta band in stance and swing phases was significantly higher in cluster 1 (stance 0.20 ± 0.04, swing 0.20 ± 0.04) than in clusters 2 (stance 0.06 ± 0.04, p < 0.05; swing 0.05 ± 0.03, p < 0.05), 4 (stance 0.10 ± 0.07, p < 0.05; swing 0.11 ± 0.07, p < 0.05) and 5 (stance 0.07 ± 0.06, p < 0.01; swing 0.07 ± 0.06, p < 0.01). The coherence of cluster 1 tended to be higher in the stance phase than in the swing phase (Fig. 3F).

## Discussion

The main purpose of the current study was to identify groups of post-stroke patients with mild motor paralysis and slow walking speed using cluster analysis to determine the association between the severity of motor paralysis and walking speed. In our study, this association suggested the existence of subgroups where walking ability could be classified according to the severity of motor paralysis and the level of walking speed in post-stroke. We were able to identify patients with mild motor paralysis and slow walking speed by cluster analysis. These findings revealed that patients with mild motor paralysis and slow walking speed cannot fully utilize the residual potential of motor function due to increased trunk instability, co-contraction of the shank muscle, and coherence. In this study, cluster 1 has been described in comparison with other clusters.

Cluster 1 had relatively mild motor paralysis; however, walking speed was still slower than other clusters. Furthermore, cluster 1 was not significantly different in FMS from cluster 4; therefore, we believe that walking speed declined due to other factors from motor paralysis. In addition, the symmetry index of single-leg support and the trailing limb angle were low. Thus, cluster 1 may have been strongly dependent on a compensatory strategy and sufficient propulsive force in the push-off phase may not have been produced^7,28^. Compensatory strategy is important for overcoming dysfunction, and cluster 1 was considered to exhibit excessive compensatory strategy because the severity of motor paralysis was mild-to-moderate. Increased instability during walking in cluster 1 appeared to limit walking speed^38^. Several causes of trunk instability were considered, including trunk dysfunction or a compensation strategy of leg dysfunction^38^. Given that cluster 1 had mild-to-moderate FMS, high trunk instability seemed to be a result of trunk dysfunction. Also, the AC of trunk acceleration reflected the regularity of each gait cycle; moreover, the AC of the anterior–posterior component was not dependent on walking speed^39^. In addition, FMS in cluster 1 and 4 were not different; however, we considered that the AC of the anterior–posterior component in cluster 1 was lower and balancing during walking was poorer than those in cluster 4. Also, cluster 1 had increased CI of TA and MG in single-leg support phase, and an increased CI was reported to be associated with a decrease in the ability to balance^40^. We considered that cluster 1 with low AC of the anterior–posterior components compensated for trunk instability by increasing the rigidity of the ankle joint during stance phase. However, the co-contraction also restricts expansion of the trailing limb angle which inhibits the ankle rocker function in the stance phase^12,41,42^, resulting in a conceivable restriction of walking speed.

To identify the descending neural drive for co-contraction, walking control was investigated using intramuscular coherence analysis. Coherence of the beta band reflected the corticospinal activity, which enabled the determination of the amount of voluntary control while walking^17,43,44^. Therefore, we consider that the increased coherence of cluster 1 indicates a high voluntary control compared to the automatic control. Our results indicate that excessive co-contraction of the TA and MG limit the trailing limb angle, and the increase in coherence may reduce walking speed. Accordingly, patients with mild motor paralysis despite excessive cortical control may mask the residual potential of motor function.

The present study had several limitations. First, we did not include other information regarding the hip and knee joints during walking. Although ankle joint functions have been reported to have a greater influence on balance than functions of the hip and knee joints post-stroke, the influence of these joints was unclear^45^. Secondly, participants used an ankle foot orthosis on the paralyzed side during walking measurements. As the force exerted by the orthosis was set to a minimum, we considered that the influence of the orthosis for walking performance was exceedingly small. However, the use of ankle foot orthosis for all participants may have been beneficial in measuring walking in patients with poor walking ability. Therefore, it was important to note that the results of this study were the outcomes in post-stroke patients who were using ankle foot orthosis. In addition, although trunk acceleration and electromyogram may be dependent on walking speed, this study did not consider walking speed in its analysis. The spatiotemporal factors are not representative of factors that are independent of changes in walking speed. Thus, these spatiotemporal factors may not give insight into the true underlying mechanism. The treadmill was not used because this study focused on the identification of subgroups based on comfortable walking speed. This was because the treadmill has different spatiotemporal parameters compared with overground walking^46^. Finally, it should be noted that the intramuscular coherence analysis conducted in this study analyzed the neural pathways involved in both TA and MG. Nevertheless, the walking speed was influenced by different factors and did not reflect linear relationships with the severity of motor paralysis. Furthermore, walking speed could characterize the pathological factors related to walking disturbances post-stroke. Characteristics of cluster 1 were considered to show an excessive compensatory strategy despite only mild motor paralysis, and further compensating and voluntary control for instability during walking by co-contraction of the lower leg muscles resulted in a decrease in walking speed. Those results may explain in part why some patients respond to traditional walking training while others do not. In the future, we must investigate the differences in balance, muscle activity pattern, and walking control and clarify factors involved in the improvement of walking speed in each cluster.

## Conclusion

Motor paralysis severity and walking speed are not linearly related; however, walking speed may be influenced by different pathological factors. Some subgroups diverged from the association of the severity of motor paralysis and walking speed. Especially, trunk instability and the co-contraction of the shank muscles were differently controlled in the stance phase of patients with mild motor paralysis and poor walking speed. Furthermore, patients in which mild motor paralysis with poor walking speed had high intramuscular coherence of the beta band in the shank muscle, thus excessive cortical control may have masked the residual potential of motor function. The detailed classification of walking disturbances based on the association between the severity of motor paralysis and walking speed will be useful for strategizing an appropriate intervention according to each pathology’s characteristics. In walking training for patients with mild motor paralysis and slow walking speed, intervention in instability and excessive cortical control may be effective.

## Data Availability

Data in this study are available to all other authors.
